# Separate Neural Systems Value Prosocial Behaviors and Reward: An ALE Meta-Analysis

**DOI:** 10.3389/fnhum.2019.00276

**Published:** 2019-08-16

**Authors:** Haixia Wang, Jian Zhang, Huiyuan Jia

**Affiliations:** ^1^School of Management, Jinan University, Guangzhou, China; ^2^Shenzhen Mental Health Centre, Shenzhen, China; ^3^College of Business Administration, Capital University of Economics and Business, Beijing, China

**Keywords:** reward, ALE, fMRI, social heuristic hypothesis, prosocial behaviors

## Abstract

**Background:** It has been argued that prosocial behaviors and momentary rewards activate similar reward systems. However, a recent theoretical hypothesis encourages a fundamentally different view. Specifically, the social heuristic hypothesis posits that individuals internalize prosocial behaviors that are advantageous in their daily social life. These advantageous behaviors are fundamentally different from tangible and immediate reward.

**Objectives:** Our objectives are to test a hypothesis that these advantageous prosocial behaviors are so critical to survival that it is necessary to have a neural system in the brain that leads people to maintain repeated social interactions. These neural systems are different from the computations of rewards because prosocial behaviors are not advantageous if only considering the computations of rewards.

**Methods:** To deepen the understanding of the neural systems of prosocial behaviors and reward, we conducted activation likelihood estimation (ALE) to examine brain activation in prosocial behaviors and reward tasks.

**Results:** Prosocial behaviors specifically activated distinct brain systems to a greater degree than reward. These systems were implicated in the processing of social behaviors and included the insula, temporal lobe, and superior temporal gyrus. By contrast, reward specifically activated the lentiform nucleus, thalamus, caudate nucleus, parahippocampal gyrus, and anterior cingulate cortex, which are associated with the brain reward system.

**Conclusions:** These findings suggest that prosocial behaviors are different from reward and involve specific brain mechanisms.

## Introduction

Prosocial behaviors refer to “a broad category of acts that are defined by some significant segment of society and/or one's social group as generally beneficial to other people” (Penner et al., 2005, p. 366). How prosocial behaviors arise is one of the fundamental questions of social life (Darwin, 2009). Prosocial behaviors, such as cooperation and altruism, are beneficial for group survival. However, these group advantageous behaviors come at a cost to the individual's momentary reward (Wilson and Wilson, 2007). It remains unclear whether the intangible advantageous benefits of prosocial behaviors are integrative or segregated to the disadvantageous computations of tangible rewards for prosocial behaviors.

Research has suggested that a positive reward accompanied by prosocial behaviors usually involves two pathways. One boosts the performers' inclusive fitness reward and leaves them with a larger number of genetically related offspring by helping their relatives (known as “kin selection theory”) (Hamilton, 1964). Another receives reciprocity reward directly from the beneficiary (Trivers, 1971) or indirectly from other observers through an established reputation (Nowak and Sigmund, 2005). Indeed, theoretical and empirical evidence has argued that prosocial behaviors produce reward and value for the performers *per se* (Harbaugh et al., 2007; Aknin et al., 2013).

Despite this progress, key questions about the nature of prosocial behaviors remain unresolved. Perhaps the most fundamental question is whether prosocial behaviors and reward are segregated or integrated in a common region in the brain. Scholars have proposed an integrative hypothesis (Landreth and Bickle, 2008; Leknes and Tracey, 2008; Levy and Glimcher, 2012), which posits that a common currency in the brain is a way to represent the value of tangible rewards and provides a common scale to value fundamentally incommensurable goods or behaviors. According to this view, the intangible advantageous benefits of prosocial behavior would be integrative to the cost of tangible reward (Saxe and Haushofer, 2008).

Although the integrative hypothesis remains highly influential, new empirical evidence has shown that prosocial behaviors may be fundamentally different from reward. This idea is consistent with new findings that prosocial behaviors may be intuitive. For example, individuals who make their decisions more quickly are more cooperative in the public goods game; moreover, forcing individuals to decide quickly increases their contributions in the game (Rand et al., 2012). Meta-analysis has also shown that intuition promotes cooperation relative to reasoning (Rand, 2016). This evidence suggest that prosocial behaviors is beyond reward-cost computations. In other words, there is a growing recognition that prosocial behaviors may have a fundamentally different nature from pure reward-cost computations. Moreover, at the theoretical level, the social heuristics hypothesis (SHH) posits that prosocial behaviors are intuitive because prosocial behavior heuristics are developed in daily social interactions where prosocial behaviors are advantageous (Rand et al., 2013). This advantage hypothesis of social behaviors implies that these group advantageous behaviors may have separate neural systems that process intangible advantageous representations from the tangible reward.

These competing hypotheses make it difficult to obtain a clear understanding of the neural systems of prosocial behaviors in the brain (Penner et al., 2005; Levy and Glimcher, 2012; Dovidio et al., 2017; Lamm et al., 2019). First, the heterogeneity of the theoretical and empirical results is partly due to the different experimental paradigms that have aimed to answer diverse aspects of prosocial behaviors (Penner et al., 2005). Second, it is unknown whether activation patterns reflect processes that are common to both prosocial behaviors and reward or instead serve incidental functions (Cutler and Campbell-Meiklejohn, 2019). Although a new theory has proposed to differentiating prosocial behaviors from pure benefit–cost computations (Rand et al., 2013), consistent neural evidence is lacking.

Thus, it is important to pool prior studies together to probe whether prosocial behaviors are fundamentally different from reward computations. In this research, we examine this segregationist model. We neither attempt a comprehensive and exhaustive discussion nor provide a detailed overview of prosocial behavior, phenomena that have been the topic of other recent reviews (Reddy and Roy, 2019). Based on the SHH, we challenge the claims of integration that prosocial behaviors are just a form of reward (Sommerville et al., 2018).

As the size and scope of the functional magnetic resonance imaging (fMRI) literature have burgeoned, it has become increasingly difficult to synthesize new data into existing competing frameworks and theories. This problem is particularly serious when trying to probe data from different domains, such as prosocial behaviors and reward. This difficulty can be solved by employing a new approach for performing coordinate-based meta-analyses (CBMAs) (Eickhoff et al., 2009; Laird et al., 2009). CBMA provides an opportunity to evaluate whether imaging studies of prosocial behaviors and reward are integrative at the neural level. The results from CBMAs provide evidence for colocalization or segregation of prosocial behaviors and reward in the brain.

## Method

### Literature Search and Study Selection

Neuroimaging studies published from January 1, 1997 to November 1, 2018, were identified by a literature search of PubMed (http://www.pubmed.org), BrainMap (http://www.brainmap.org/software.html#Sleuth), and Google Scholar (https://scholar.google.com.hk/) for different combinations of the terms “fMRI,” “neural,” “reward,” “money,” “value,” “prosocial,” “altruis^*^,” “charity,” “charitable,” “public goods,” “cooperation,” “public goods,” “social value orientation,” “reputation,” “dictator,” “ultimatum,” “trust game,” and “prisoner^*^.” Further papers were obtained by reference tracing of the retrieved papers and previous meta-analyses on prosocial and reward. Papers were considered if they reported novel fMRI data not reported elsewhere, collected while participants conducted tasks regarding prosocial behaviors and reward, and analyzed whole-brain data.

To provide comprehensive, best-practice analyses of consistent activation by prosocial behaviors and reward, we applied the inclusion and exclusion criteria discussed below.

1. To infer consistency across experiments, only fMRI studies were included.
2. To ensure that the likelihood of brain activation under the null hypothesis is equal across the brain (Eickhoff et al., 2009), experiments were only included if they reported whole-brain activation coordinates.
3. Experiments were only included if reported coordinates were represented in standardized space, either Montreal Neurological Institute (MNI) coordinates or Talairach (TAL) coordinates. If coordinates were localized in TAL, they were converted to MNI space employing software embedded in GingerALE (Eickhoff et al., 2009).
4. Coordinates were only included if they were the result of a contrast analysis that directly tested prosocial behaviors or reward.
5. Finally, only experiments that used healthy participants were included to help control for individual differences in brain activation across populations.

After the exclusion and inclusion criteria had been applied, a total of 114 experiments consisting of 2,023 participants and 1,273 foci were used (see Figure S1). Prosocial behaviors included 136 foci from 19 experiments with 361 participants. Reward included 1,137 foci from 95 experiments with 1,662 participants.

Separate meta-analyses were only performed if a sufficient number of experiments were available (>17 experiments) (Eickhoff et al., 2016). All analyses (except those with <17 experiments) were repeated to examine (1) patterns of common and specific activation across prosocial behaviors and reward, and (2) corrected results.

### Activation Likelihood Estimation

Following previous studies, the activation likelihood estimation (ALE) meta-analyses were conducted according to the standard procedures employing GingerALE 2.3.6 software (Eickhoff et al., 2009). Coordinates reported in TAL were transformed into Montreal Neurological Institute (MNI) space by using TAL-MNI conversion software, embedded within GingerALE. All results in our meta-analyses were thresholded at a cluster-level corrected threshold of *p* < 0.05 (cluster-forming threshold at voxel level *p* < 0.001).

## Results

### Meta-Analyses Across Prosocial Behavior Experiments

**Figure 1:**
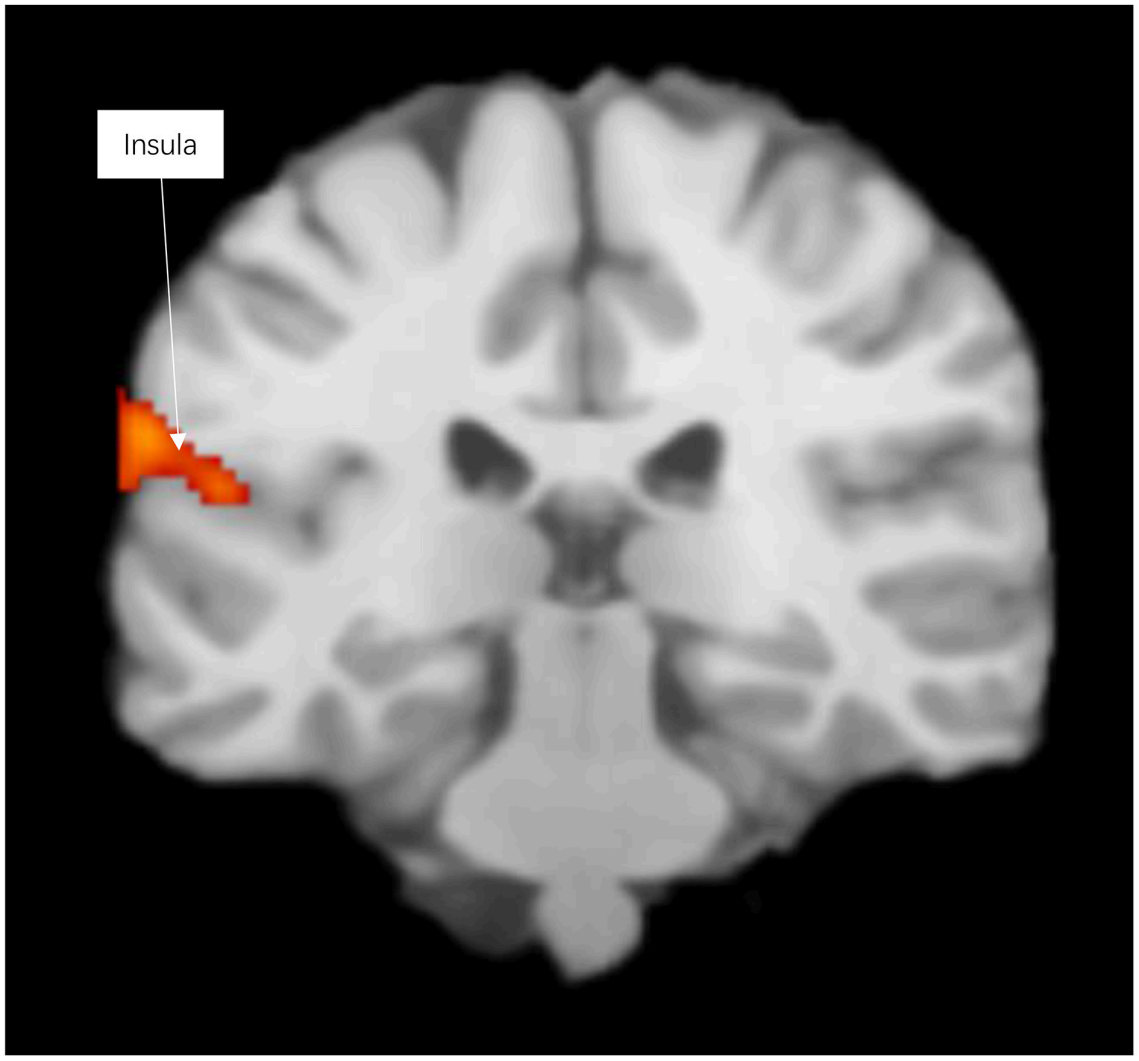
Significant clusters in the ALE meta-analysis across prosocial behavior experiments (cluster-level corrected threshold of *p* < 0.05).

Prosocial behaviors activated emotional prosocial brain, the insula, the temporal lobe, and the superior temporal gyrus (see Figure 1 and Table S1).

### Meta-Analyses Across Reward Experiments

**Figure 2:**
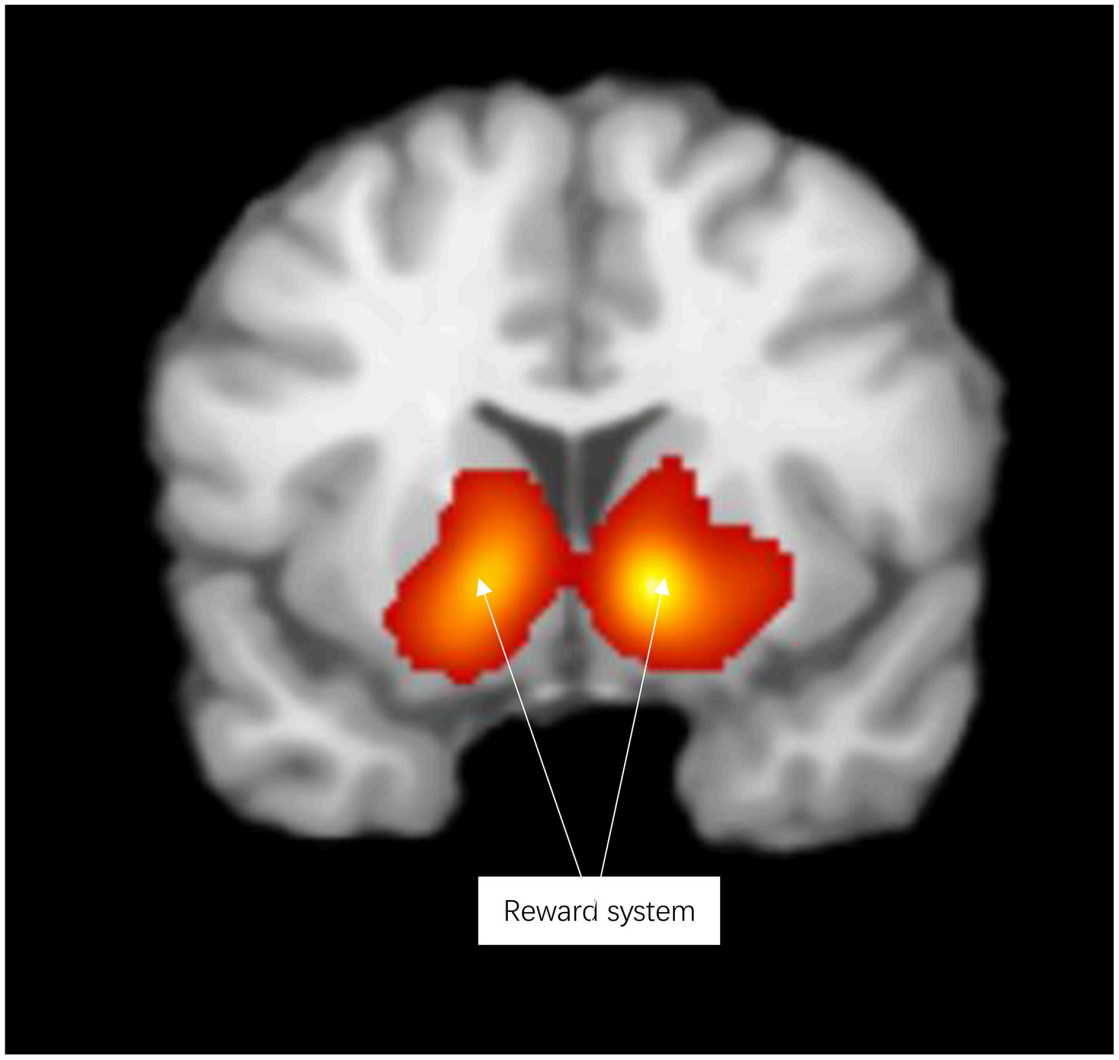
Significant clusters in the ALE meta-analysis across reward experiments (cluster-level corrected threshold of *p* < 0.05).

Reward activated the reward system, including the lentiform nucleus, thalamus, caudate nucleus, anterior cingulate cortex, parahippocampal gyrus, inferior frontal gyrus, and medial frontal gyrus (see Figure 2 and Table S2).

### Contrast Meta-Analyses

Contrasting the activation caused by prosocial behaviors and reward, we found that prosocial behaviors significantly activated the insula to a greater degree (see Figure S2 and Table 1). The lentiform nucleus, thalamus, caudate nucleus, parahippocampal gyrus, and anterior cingulate cortex showed greater activation with reward than with prosocial behaviors (see Figure S3 and Table 2).

**Table 1:** Brain areas specifically activated by prosocial behaviors from ALE analysis.

**Region**	**BA**	**MNI coordinates**		
		***X***	***Y***	***Z***
L Superior temporal gyrus	22	−63	−38	21
L Temporal lobe	42	−62	−33	24
L Insula	13	−58	−31	20

*Clusters in the contrast analyses were thresholded at uncorrected p <0.05 with 5,000 permutations and a minimum cluster size of 50 mm^3^*.

**Table 2:** Brain areas specifically activated by reward from ALE analysis.

**Region**	**BA**	**MNI coordinates**		
		***X***	***Y***	***Z***
R Parahippocampal gyrus		−8	7	−1
R Lentiform nucleus		24	20	−2
R Extra-nuclear		5	4	3
R Caudate		10	26	−5
R Thalamus		4	−18	16
L Thalamus		0	−20	6
L Cingulate gyrus	32	4	30	36

*Clusters in the contrast analyses were thresholded at uncorrected p <0.05 with 5,000 permutations and a minimum cluster size of 50 mm^3^*.

We conducted conjunction analyses to identify the common areas between prosocial behaviors and reward. None of the conjunction meta-analyses revealed any significant results.

## Discussion

How does the brain compare prosocial behaviors and reward? One intriguing hypothesis is that they can represent the subjective value of all reward types on a common neural scale (Levy and Glimcher, 2012). However, does this common neural currency exist? The results from our meta-analysis refute claims that prosocial behaviors and reward are the same thing and shared common neural currency in the brain. Instead, our observations show that prosocial behaviors and reward are fundamentally different from each other. In fact, these results from our meta-analyses do not preclude the possibility that they may integrated at finer levels of analysis. For instance, it is possible that prosocial behaviors and reward may be integrated into individual participants or neurons. Similarly, common neural currency may be present on a finer timescale that is resolved by conventional fMRI studies. Nevertheless, what these results from our meta-analysis do demonstrate is that conventional fMRI studies of prosocial behaviors and reward are segregated into different brain areas.

These findings are consistent with the theory of SHH (Rand et al., 2012, 2013; Rand, 2016). This hypothesis was set out to answer an interesting question: is our first response to be selfish such that we show prosocial behaviors through careful reasoning? Or are we predisposed toward prosocial behaviors, with deliberative self-control leading to self-interest? SHH posits that individuals internalize behaviors that are typically advantageous and successful in their daily repeated social behaviors and interactions (Rand et al., 2013). These advantageous and successful prosocial behaviors are fundamentally different from tangible and immediate rewards, which is supported in our meta-analysis. Thus, our results suggest that interventions that designed to promote prosocial behaviors should be aware of the fundamental differences between prosocial behaviors and reward.

The specific brain areas involved in prosocial behaviors in our meta-analysis were mostly located in the insula. In a review of prosocial behaviors literature (Cutler and Campbell-Meiklejohn, 2019), the insula was related to prosocial behaviors. The insula may encode intangible advantageous benefits of prosocial behaviors beyond reward-cost computations.

Reward processing in our meta-analysis was distinguished by modulating activity in the lentiform nucleus, thalamus, caudate nucleus, parahippocampal gyrus, and anterior cingulate cortex. The results from previous neuroimaging studies (Shackman et al., 2011; Levy and Glimcher, 2012) and the current meta-analysis support the notion that humans independently process prosocial behaviors and reward. Importantly, segregation models and domain-specific neural implementation were threshold-dependent (Jiang and Egner, 2013); thus, probing the potential factors influencing the thresholding between prosocial behaviors and reward would be a valuable future direction.

A possible limitation of the current meta-analysis is that we did not include psychological and motivational experiences associated with prosocial behaviors, such as gratitude, awe, compassion, kindness, and empathy (Bartlett and DeSteno, 2006; Grant and Gino, 2010; Masten et al., 2011; Piff et al., 2015; Flournoy et al., 2016; Van der Graaff et al., 2018). Based on SHH, prosocial behaviors are advantageous in repeated daily life. This theory is silent on the motivational experiences associated with prosocial behaviors. Thus, it is unclear whether prosocial motivation such as compassion are also advantageous in daily life. It has been argued that empathy, compassion, and prosocial behaviors are distinct phenomena and they differ with respect to their neural mechanisms (Lamm et al., 2019). Thus, to directly test the prediction from SHH, we only included prosocial behaviors. Still, future studies may focus on whether psychological and motivational experiences that induce prosocial behaviors, such as empathy and compassion, share common neural systems with reward.

Two methodological caveats must be noted. First, as stated above, we did not collect experiments about prosocial motivation such as empathy, compassion, and kindness. We only included prosocial behaviors to obtain behavioral level experiments. Although prosocial behaviors are more directly related to our theoretical hypothesis that prosocial behaviors instead of prosocial motivation are advantageous in repeated daily social interactions, the limited experiments of these prosocial motivations may produce less specific regions of prosocial psychological experiences. Second, we used the CBMA method. It has been argued that more information may be obtained employing a map-based meta-analysis (Maumet and Nichols, 2015). However, this undertaking was not possible because the contrast and standard error maps are not widely shared in our study.

## Conclusion

We investigated the integrative hypothesis and segregationist model regarding prosocial behaviors and reward by using a meta-analytical method. Prosocial behaviors specifically activated distinct brain systems to a greater degree. These systems were implicated in the processing of social behaviors. By contrast, reward specifically activated the brain reward system. We documented that prosocial behaviors are different from reward-cost computations and involve specific brain mechanisms. These results suggest that interventions that designed to promote prosocial behaviors should be aware of the fundamental differences between prosocial behaviors and reward.

## Author Contributions

Each author made contributions to the conception, design of the work, the acquisition, analysis, and interpretation of data. HW drafted the manuscript. HJ and JZ revised it.

### Conflict of Interest Statement

The authors declare that the research was conducted in the absence of any commercial or financial relationships that could be construed as a potential conflict of interest.
