# Supplementary material for: Separate Neural Systems Value Prosocial Behaviors and Reward: An ALE Meta-Analysis
Source: Front Hum Neurosci. 2019 Aug 16;13:276. doi: 10.3389/fnhum.2019.00276 (PMC6707092; doi:10.3389/fnhum.2019.00276)
Supplement: Supplementary file 1 [file Data_Sheet_1.PDF]

Separate neural systems value prosocial behaviors and reward: An ALE meta-analysis

Supplementary Material

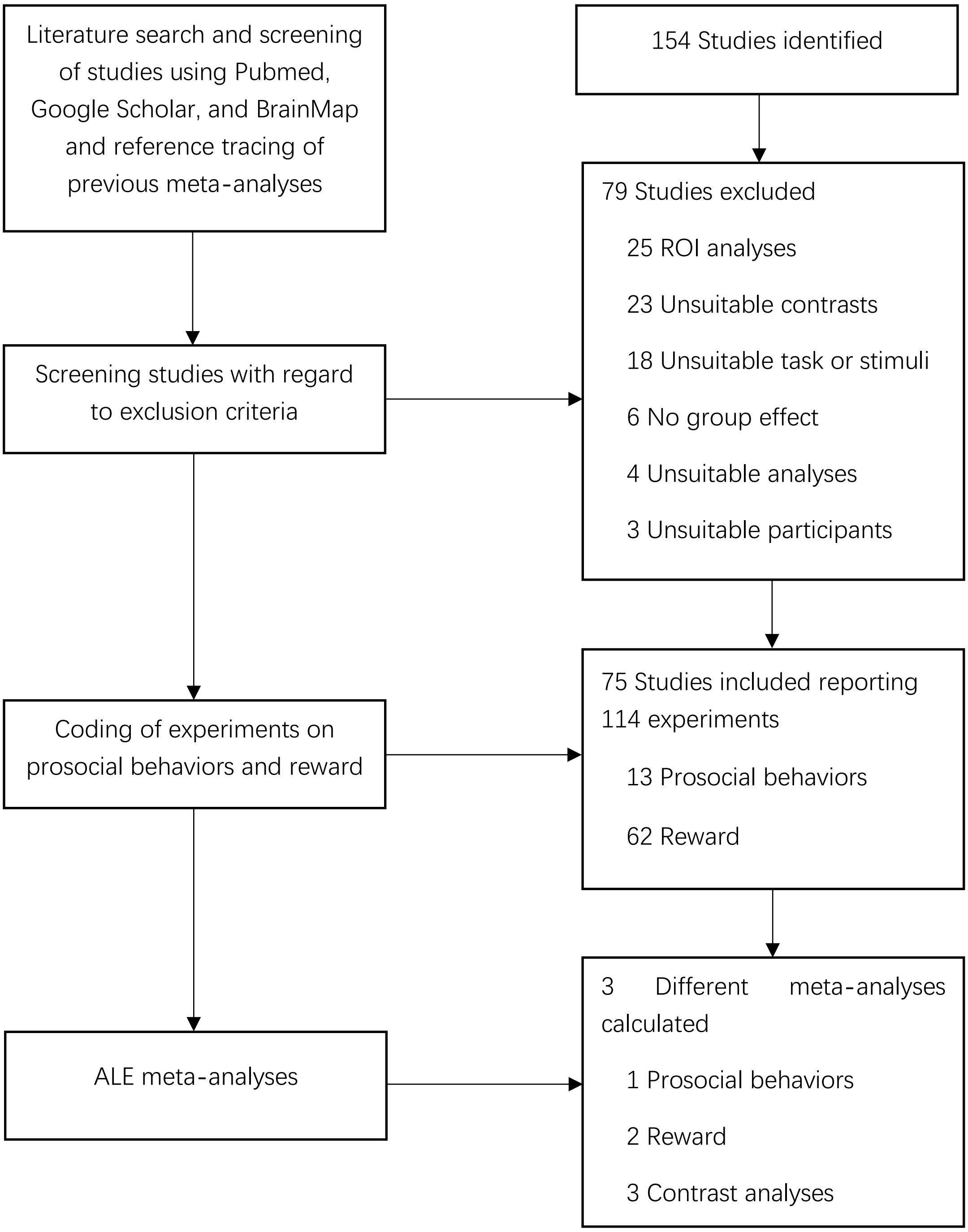

Figure S1. Flowchart of the different steps conducted.

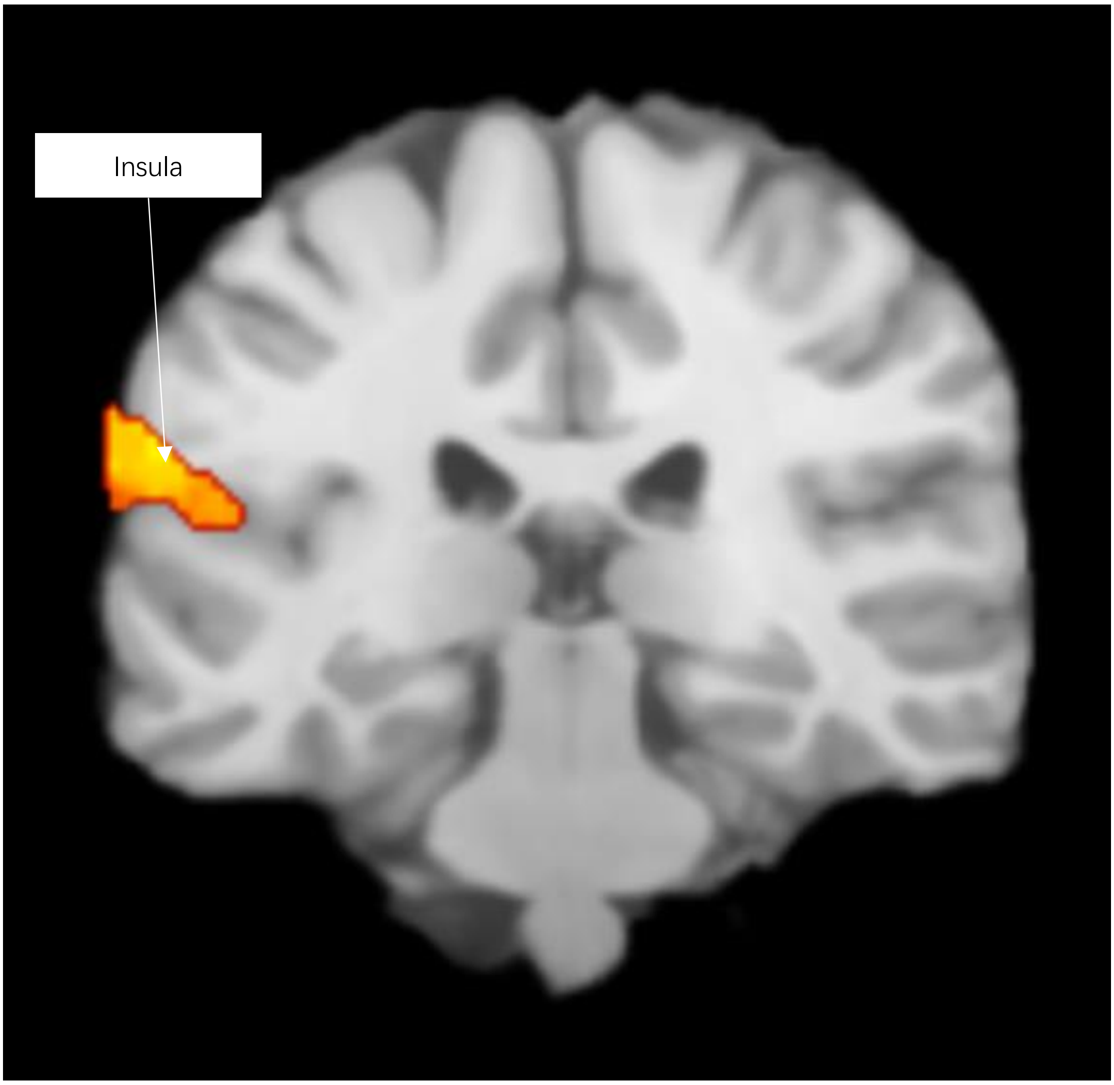

Figure S2. Significant clusters in the ALE contrast meta-analysis showing patterns of specific activation for prosocial behaviors experiments (Clusters in the contrast analyses were thresholded at uncorrected $p < 0.05$ with 5000 permutations and a minimum cluster size of $50mm^3$).

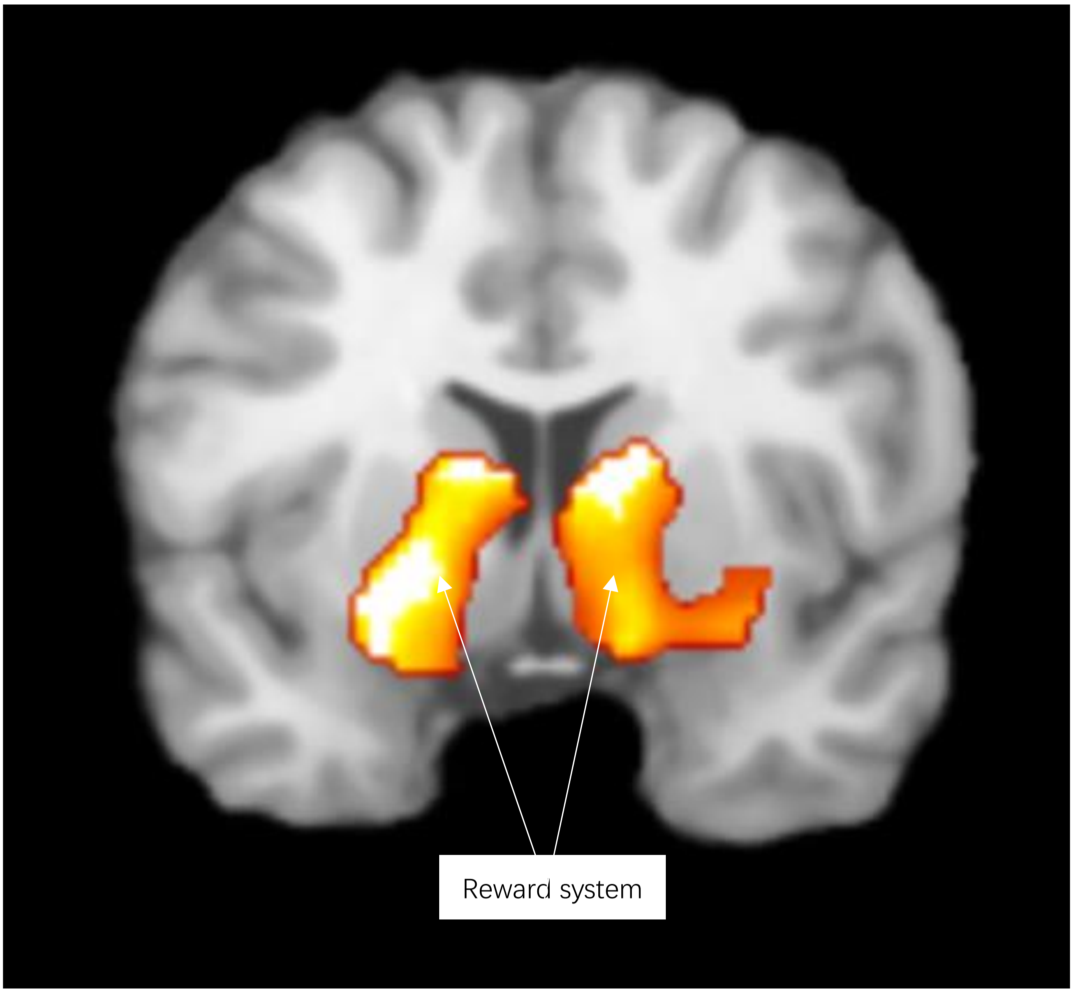

Figure S3. Significant clusters in the ALE contrast meta-analysis showing patterns of specific activation for reward experiments (Clusters in the contrast analyses were thresholded at uncorrected $p < 0.05$ with 5000 permutations and a minimum cluster size of 50mm^3).

Table S1. Brain areas activated by prosocial behaviors from ALE analysis

		MNI coordinates		
Region	BA	X	Y	Z
L Superior Temporal Gyrus	42	-64	-34	22
L Temporal Lobe	41	-50	-30	14
L Insula	13	-59	-34	20

Note. Cluster-level corrected threshold of $p < .05$.

Table S2. Brain areas activated by reward from ALE analysis

		MNI coordinates		
Region	BA	X	Y	Z
R Caudate		12	8	-4
L Lentiform Nucleus		-12	8	-4
R Claustrum		34	22	-2
L Claustrum		-32	20	-6
R Thalamus		4	-20	12
L Thalamus		-14	-10	14
L Midbrain		-6	-18	-12
L Cingulate Gyrus	32	0	30	32

Note. Cluster-level corrected threshold of $p < .05$.
